# The characteristic of Yatu morphogenesis and the efficacy of exogenous hormones on the development of Yatu during fruit development in ‘Yali’ pear (*Pyrus bretschneideri* Rehd.)

**DOI:** 10.1080/15592324.2022.2106075

**Published:** 2022-08-02

**Authors:** Ying Zhang, Wanjun Liu, Xiaoxin Shi, Yuxing Zhang, Guoqiang Du

**Affiliations:** a College of Horticulture, Hebei Agricultural University, Baoding, China; b Pear Engineering and Technology Research Center of Hebei, College of Horticulture, Hebei Agricultural University, Baoding, China

**Keywords:** Primitive cell, cell division, endogenous hormone, GA_3_+6-BA, paclobutrazol

## Abstract

Yatu is a protuberance formed on the base part of ‘Yali’ pear fruit, near the pedicel, causing a shape like a duck head termed Yatu. It is a typical phenotypic trait to evaluate fruit appearance quality. The mechanism for Yatu formation has not been clear yet. Here, 90.8% of fruits with Yatu generated in outer base part of fruits. Primitive cells of Yatu were found at 10 days after pollination (DAP). There were higher expression levels of *PbGA20ox2, PbIPT7a*, and *PbIPT5a*, lower transcription levels of *PbGA2ox1, PbNCED1*, and *PbNCED3* in outer base part of fruits at 10 DAP, accompanied with significantly higher levels of GA_3_, ZR, (GA_3_+ ZR)/ABA, and lower ABA content compared to that in the inner base part of fruits. GA_3_ + 6-BA promoted Yatu development by increasing GA_3_ content at 10 and 20 DAP, and ZR content at 20 DAP. PAC suppressed Yatu morphogenesis and development by increasing ABA level at 10 DAP. These results suggest that Yatu usually generated in outer base part of fruits, relatively higher GA_3_ and ZR contents, lower ABA content promoted Yatu morphogenesis and development.

## Introduction

‘Yali’ (*Pyrus bretschneideri* Rehd.) is native to the Hebei province in China and is one of the superior traditional pear cultivars.^1^ The fruits have a very high economy and nutrition value.^2^ Yatu is a protuberance that forms on the base of a ‘Yali’ pear and looks like a duck head. It is a typical phenotypic trait to evaluate fruit appearance quality.^3^ The phenomenon of fruits without Yatu or with non-typical Yatu often occurs in ‘Yali’ production, although the reason is not yet clear.^4^ It is important and necessary to clarify the mechanism of Yatu morphogenesis and development.

Previous studies have found the percent of first-order fruits with Yatu and typical Yatu were significantly higher than that of sixth-order fruits.^5^ The base part of proximal fruit within an inflorescence possessed relatively higher gibberellin acid (GA_3_) and zeatin riboside (ZR) content, lower abscisic acid (ABA) content, and well-developed vascular bundles, which enhanced fruit sink strength to provide sufficient assimilate for Yatu formation.^5^ While the cells in the base part of fruit that tend to develop into Yatu should be found. How the hormones functioned and controlled Yatu formation should be explicit.

In the early phase of fresh fruit development, cell division and cell expansion played a crucial role in the determination of fruit size and shape.^6,7^ The cell division of ‘Yali’ pear fruit started before bloom and ceased at about 25 days after flowering.^3^ Cell division intensity and duration affected cell number, which was the determining factor for fruit growth rate.^8^ Cell expansion depended on increasing in turgor pressure caused by accumulation of storage products and sugar.^9,10^ In pear fruit development, the specific temporal and spatial pattern of cell division and cell expansion determined the fruit phenotypic trait.

Plant hormones played a critical role in regulating cell division and cell expansion.^8^ Cytokinin acted as an inducible factor to exert initiating and promoting functions on cell division.^11,12^ It regulated the assimilate transportation and related protein synthesis to regulate cell expansion.^13^ Gibberellins promoted the longitudinal cell expansion by changing the stability of the microtubules via affecting their association with the plasma membrane.^14,15^ ABA counteracted gibberellin efficiency during fruit set and development, which emphasized the importance of hormone homeostasis during fruit development.^16,17^ The objectives of this research were to study the characteristic of Yatu morphogenesis and clarify the regulated mechanism of plant hormones on Yatu development.

## Materials and methods

### Plant materials and treatment

**Figure 1.**
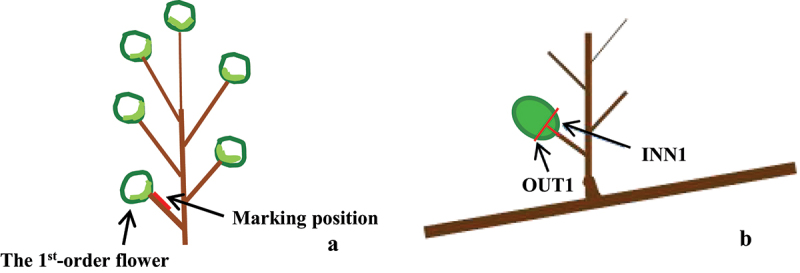
The diagram of pear inflorescence. (a) The left black arrow indicates the first-order flower/fruit on an inflorescence. The red straight line indicates the position for marking inner side surface of the peduncle with a dissecting needle. (b) Tissues sampled from the inner and outer base part of the first-order fruit were named INN1 and OUT1, respectively. The red lines indicate cutting position for sampling.

The experiment was carried out in 2018 in a commercial pear orchard in Botou, Hebei province, China (116.53E, 38.15 N). ‘Yali’ (*Pyrus bretschneideri* Rehd.) trees on *P. betulaefolia* rootstock were planted in 1988 with a spacing at 4 m × 6 m and a north-to-south row orientation. At day 2 before bloom (DBB), the first-order flower was bagged, and the others within the same inflorescence were thinned (Figure 1a). During bloom period, a controlled pollination was made using the prepared pollen of ‘Cuili’, which is a native pear variety. As pollinating, a marker was made on inner side surface of the peduncle that closed to the base part of fruit to help us easily recognize inner and outer base part of fruit while the fruit growing and further drooping (Figure 1a). The percent of fruits with Yatu that waas generated in the inner and outer base parts of fruits was counted. Samples that were collected from inner and outer base part of fruits were named INN1 and OUT1, respectively (Figure 1b). They were collected at 2 DBB and 10, 20, and 35 days after pollination (DAP) for determining GA_3_, ZR, and ABA content. Samples selected at 10 DAP were used for preparing paraffin section to observe cell morphology and determe the relative expression levels of genes related to hormone biosynthesis and metabolism. The experiment was designed as a randomized complete block with three replications of three trees each.

### Plant growth regulators treatment

Spreading GA_3_+6-BA and PAC were separately applied on two groups of fruits to observe the function on Yatu morphogenesis and development. The application concentrations were 300 mg/L for GA_3_ and 6-BA, 4000 mg/L for PAC, which have been testified as optimal concentrations. The base part of fruit, approximately a circle of 1/5 the length of a fruit, was treated by hormones. GA_3_ + 6-BA and PAC were both applied at 2 DBB and 10 DAP, respectively. GA_3_ and PAC were dissolved in ethanol to make stock solution, 6-BA was dissolved in 1 M NaOH to make stock solution. The control was distilled water containing the same concentration of ethanol and NaOH as that used in treatment groups. One hundred pears of each treatment were randomly selected for observing Yatu development. INN1 and OUT1 that sampled at 2 DBB, 10, 20, and 35 DAP were used for determining GA_3_, ZR, and ABA contents. INN1 and OUT1 that sampled at 10 DAP were used for determining the relative expression levels of genes related to hormone biosynthesis and metabolism. The experiment was designed as a randomized complete block with three replications of three trees each.

### Classification criteria for Yatu

**Figure 2.**
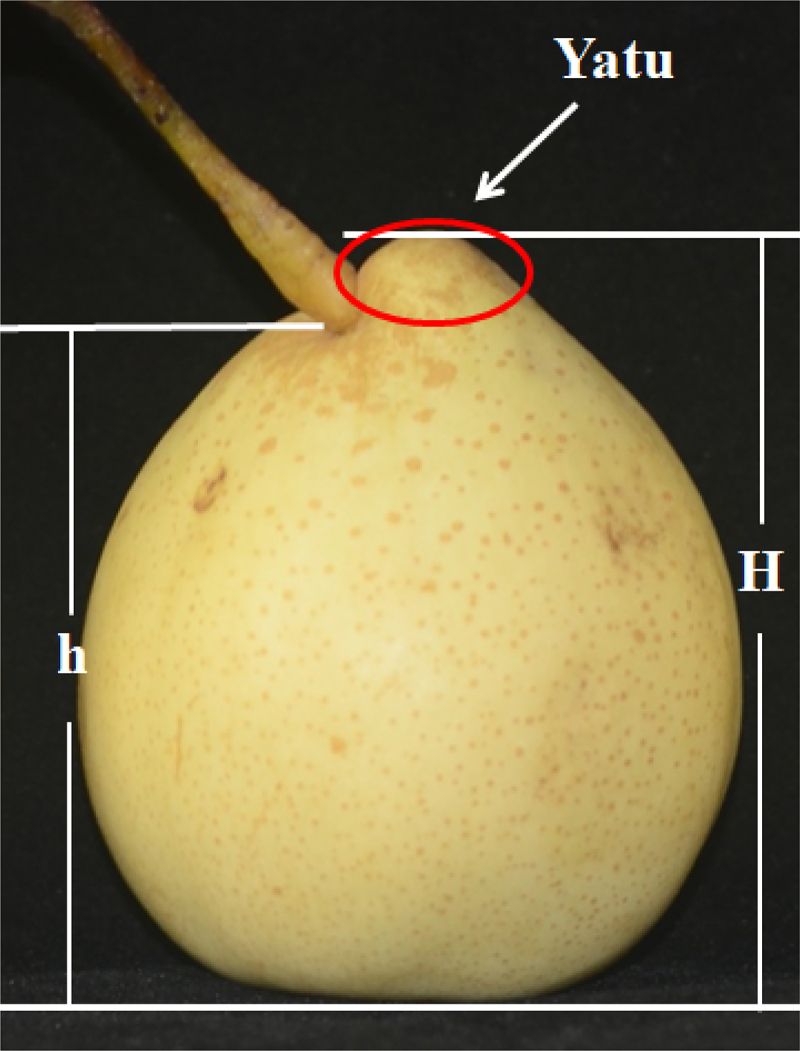
Yatu, which is circled by the red oval, developed well. The height of the right and left side of the fruit is indicated by H and h, respectively. The classification of Yatu was determined by the difference between H and h (e.g. H-h).

The difference of a fruit height between two opposite sides was used as a grading standard for Yatu. The height of one side with Yatu was showed by H, the height of opposing side was showed by h. The classification of Yatu was determined by the difference between H and h (e. g. H-h) (Figure 2). Difference that reached 10 mm or more was defined as typical Yatu. Difference no less than 5 mm and lower than 10 mm was defined as non-typical Yatu.

### Investigation of fruits with different types of Yatu

Mature fruits were harvested at 162 DAP. One hundred mature fruits of each treatment were randomly selected and examined. The test was replicated three times.

### Preparation for paraffin section

Samples were kept in a formaldehyde acetic acid alcohol (FAA) fixative first. The paraffin sections were prepared as described by ^18^. They were dehydrated by an ascending concentration of ethanol, cleaned by dimethylbenzene, embedded in paraffin, sectioned on a microtome, double stained by sarranine and fast green, rehydrated, and observed and photographed under a microscope.

### Hormone extraction, purification, and quantification

The method of hormone determination was done as described by ^5^. Samples were ground to powder in liquid nitrogen. Hormones were extracted in 80% methanol containing 0.2 g butylated hydroxytoluene under ultrasonic wave, concentrated under nitrogen gas, redissolved in methanol, and filtrated through 0.22 µm filter membrane. High-performance liquid chromatography (HPLC) was used to determine the concentration of GA_3_, ZR, and ABA.

### RNA extraction and quantitative real-time PCR

Total RNA was isolated with RNAprep Pure Plant Kit (Tiangen, Beijing, China). Reverse transcription was performed with FastQuant RT Kit (Tiangen, Beijing, China). *PbGAPDH* was used as the internal control.^19–21^All specific primers were designed on NCBI website and further confirmed by the corresponding melting curves with a single sharp. All primer sequences used for RT-qPCR are listed in Table 1. Quantitative real-time PCR was performed in three replicates using the TransStart Top Green qPCR SuperMix (Transgen, Beijing, China) on a Bio-Rad CFX-96 Real-time PCR Detection System. The procedure was set as follows: template initial denaturation at 95°C for 30 s, template denaturation at 95°C for 15 s, primer annealing at 60°C for 15 s, 40 cycles extension at 72°C for 30 s, and followed by the melting curve analysis. The relative gene expression levels were calculated and normalized by the 2^−ΔΔCt^ method.^22^

**Table 1.** Primers used for qRT-PCR.

Gene name	Primer sequences	
*PbGAPDH*	qPbGAPDH-F	5’-GGTCAAGCATCTTTGACGCC-3’
	qPbGAPDH-R	5’-ACCGAGGGAGCATTTAGTCAC-3’
*PbGA2ox1*	qPbGA2ox1-F	5’-TCTTGAGCTGATGTCTGATGGA-3’
	qPbGA2ox1-R	5’-CTGGCACTGGCACTACTCAA-3’
*PbGA20ox2*	qPbGA20ox2-F	5’-ACGGGAATTACAAGAGCGGG-3’
	qPbGA20ox2-R	5’-GCGGCTTCACGACTTTATCG-3’
*PbGA3ox1*	qPbGA3ox1-F	5’-ACAGCGTAAAAGTCGGCTAGA-3’
	qPbGA3ox1-R	5’-CCAAGAACATGGCCAACCAA-3’
*PbIPT3*	qPbIPT3-F	5’-GACCTAGCCACCTGTTTCCC-3’
	qPbIPT3-R	5’-CCCTAGCAAATGGTGCGGTA-3’
*PbIPT5a*	qPbIPT5a-F	5’-GGAAACGAATCAAGCGTGGG-3’
	qPbIPT5a-R	5’-TGGTAGTGGCGGTGATGTTC-3’
*PbIPT5b*	qPbIPT5b-F	5’-AAGTAGGTCTGGCCGTCTCT-3’
	qPbIPT5b-R	5’-GACAGCAGCGTTTCCATTCC-3’
*PbIPT7a*	qPbIPT7a-F	5’-GCTGGCGGATCCAACTCTTA-3’
	qPbIPT7a-R	5’-CAGGCAACGACACATCAAGC-3’
*PbNCED1*	qPbNCED1-F	5’-TGACCAAACCATGCCTTCCA-3’
	qPbNCED1-R	5’-AAGAAAGGTGGCGGTGTCTT-3’
*PbNCED3*	qPbNCED3-F	5’-ATCTGATATGCCCCCGGAGA-3’
	qPbNCED3-R	5’-GGGTGTCCCAAACCTCTCAC-3’

### Statistical analysis

Data Processing System (DPS®) software was used to analyze the experimental data. The significance of differences among mean values was determined by *t-test* (*p*≤ .05) and Duncan’s multiple-range test (*p*≤ .05) using least significant ranges means.

## Results

### Yatu usually generated in outer base part of fruit

**Figure 3.**
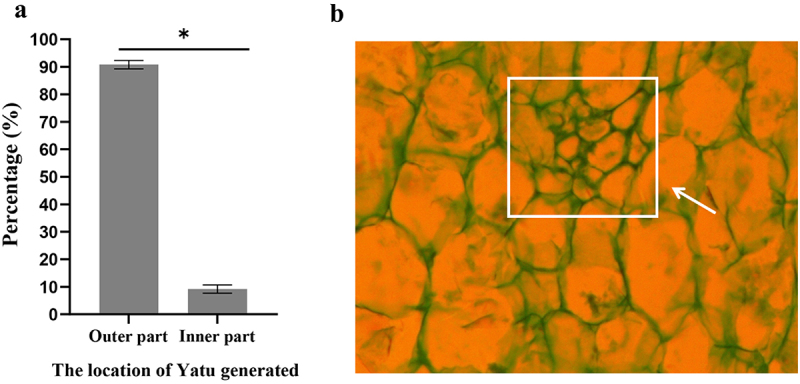
The location of Yatu generated and the primitive cell morphology (magnification 400×). (a) The percent of fruits generated in the inner and outer base parts of fruits. Asterisks above the bars indicate a significant difference (*t*-test, *p*≤ .05). (b) Primitive cells of Yatu are indicated by the white arrow.

The percent of fruits with Yatu generated in outer base part of fruits was 9.9 times higher than that in inner (Figure 3a). A lot of parenchyma cells that were characterized by small cells with more layers gathered in outer base part of fruits were found at 10 DAP. They were called primitive cells of Yatu. The 10 DAP was considered as the strongest cell division stage for Yatu. Around the primitive cells, other parenchyma cells were large and loosely arranged (Figure 3b).

### Hormone contents and proportions differed in inner and outer base part of fruits

**Figure 4.**
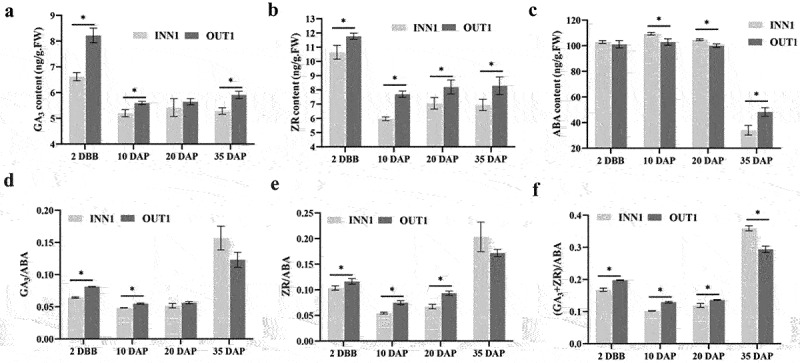
Hormone contents and the ratio between them in the inner and outer base parts of fruits. (a) Gibberellin acid (GA_3_). (b) Zeatin riboside (ZR). (c) Abscisic acid (ABA). (d–f) GA_3_/ABA, ZR/ABA, and (GA_3_+ ZR)/ABA. Asterisks above the bars indicate significant differences (*t*-test, *p*≤ .05) .

The GA_3_ content in the outer base part of fruits was significantly higher at 2 DBB, 10 DAP, and 35 DAP, and showed no difference at 20 DAP from the inner base part of fruits (Figure 4a). The contents of ZR in the outer base part of fruits all were significantly higher than that in the inner base part of fruits (Figure 4b). ABA content in outer base part of fruits was significantly lower at 10 DAP and 20 DAP compared to that in inner (Figure 4c). At 2 DBB and 10 DAP, the ratio of GA_3_/ABA, ZR/ABA, and (GA_3_+ ZR)/ABA in the outer base part of fruits was significantly higher than that in inner (Figure 4 d-–f). Consistent with Yatu usually generated in the outer base part of fruits, the higher content of GA_3_, ZR, (GA_3_+ ZR)/ABA, the lower content of ABA in the outer base part of fruits promoted Yatu formation.

### Expression levels of genes related to the biosynthesis and metabolism of GA, cytokinin, and ABA in the inner and outer base part of fruits

**Figure 5.**
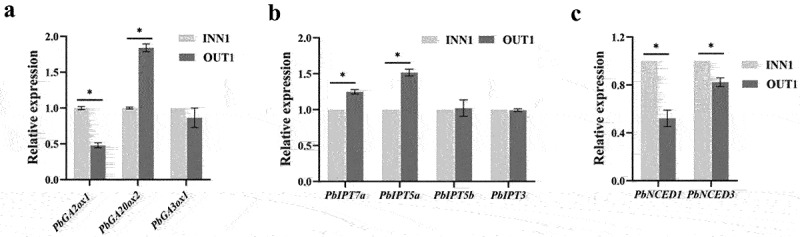
Expression patterns of gibberellin acid (GA) metabolism (a), cytokinin (CTK) biosynthesis (b), and abscisic acid (ABA) biosynthesis-related genes (c) in the inner and outer base parts of fruits at 10 days after pollination. Asterisks above the bars indicate significant differences (*t-*test, *p*≤ .05) .

The relative expression level of *PbGA2ox1* in the inner base part of fruits was 2.1 times higher than that in the outer base part of fruits. The gene *PbGA20ox2* possessed relatively higher expression level in the outer base part of fruits than in the inner base part of fruits. There was no significant difference in *PbGA3ox1* expression level between the inner and outer base parts of fruits (Figure 5a). The relatively lower *PbGA2ox1* transcription level and higher *PbGA20ox2* transcription level in the outer base part of fruits were consistent with higher GA_3_ accumulation at 10 DAP (Figure 4a).

The transcription levels of *PbIPT7a* and *PbIPT5a* in the outer base part of fruits were significantly higher than that in the inner base part of fruits (Figure 5b). It was consistent with higher ZR content in the outer base part of fruits at 10 DAP (Figure 4b). Both *PbIPT5b* and *PbIPT3* expression showed no significant differences between the inner and outer base parts of fruits (Figure 5b).

As shown in Figure 5c, the genes *PbNCED1* and *PbNCED3* possessed relatively lower expression levels in the outer base part of fruits than that in the inner base part of fruits. It was consistent with the relatively lower ABA content in the outer base part of fruits (Figure 4c).

### Exogenously applied GA_3_ + 6-BA and PAC changed the ability of fruits to form Yatu

**Figure 6.**
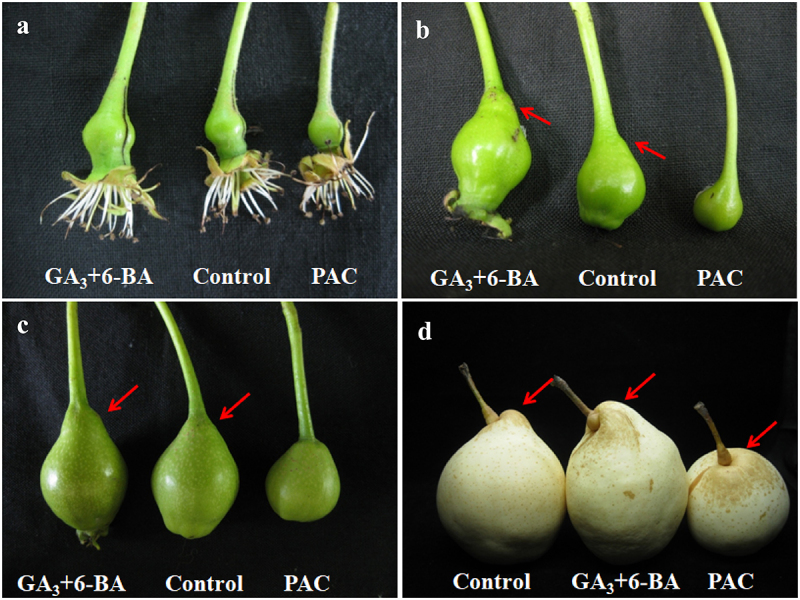
The influence of GA_3_ + 6-BA and PAC on Yatu morphogenesis and development, including 10 (a), 20 (b), 35 (c), and 162 days after pollination (d), respectively. In (a–c), the left and right fruit were treated by GA_3_ + 6-BA and PAC, respectively, the middle fruit was control. In (d), the left fruit was control, the middle and right fruits were treated by GA_3_ + 6-BA and PAC, respectively. The red arrows indicate Yatu in different developmental stages.

At 10 DAP, Yatu could not be seen on the base part of all treated and untreated fruits (Figure 6a). At 20 DAP, the phenotype of Yatu obviously appeared in GA_3_ + 6-BA-treated fruits and control fruits. The size of Yatu was much bigger in GA_3_ + 6-BA-treated fruits than control fruits. There was still no Yatu generated in fruits treated by PAC (Figure 6b). At 35 DAP, the size of Yatu in GA_3_ + 6-BA treated fruits and control fruits increased with time extension. There was still no Yatu appearing in PAC-treated fruits (Figure 6c). At 162 DAP (Figure 6d), the size of Yatu treated by GA_3_ + 6-BA was significantly higher than that of control fruits, a small bump appeared on the base part of fruit treated by PAC.

After harvest, the percent of fruits with different types of Yatu was counted (Table 2). There was no significant difference between GA_3_ + 6-BA-treated and control fruits on total percent of fruits with Yatu. GA_3_ + 6-BA significantly increased the percent of fruits with typical Yatu. Yatu did not appear in PAC-treated fruits. Altogether, the results strongly suggest that GA_3_ + 6-BA promoted the development of Yatu and PAC showed the opposite efficacy.

**Table 2.** The influence of GA_3_ + 6-BA and PAC on constitution of different types of Yatu.

Application time	Treatment	Total percent of fruits with Yatu			Non-typical Yatu		Typical Yatu	
					5 mm≤H-h < 10 mm		H-h ≥ 10 mm	
2 DBB+10 DAP	GA_3_ + 6-BA	92.22	a	26.56		b	65.66	a
	PAC	0	b	0		c	0	c
	Distilled water	92.15	a	44.69		a	47.46	b

The different letters following the values in the same column indicate a significant difference (*p*≤ .05).

### Endogenous hormone contents in base part of fruits treated by GA_3_ + 6-BA and PAC

**Figure 7.**
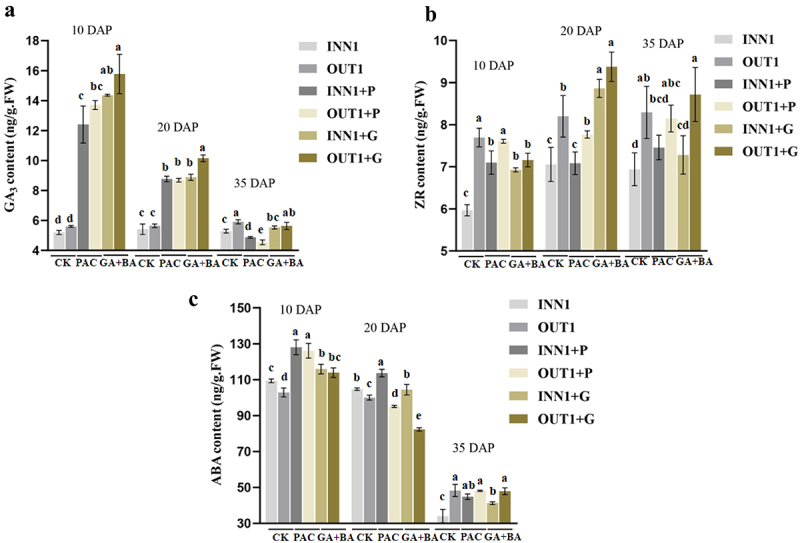
The influence of GA_3_ + 6-BA and PAC on hormone contents in the inner and outer base parts of fruits at 10, 20, and 35 days after pollination. (a) Gibberellin acid (GA_3_). (b) Zeatin riboside (ZR). (c) Abscisic acid (ABA). Different letters above the bars indicate significant difference (Duncan’s multiple-range test, *p*≤ .05). INN1/OUT1 + P, the inner/outer base part of fruits treated by PAC; INN1/OUT1 + G, the inner/outer base part of fruits treated by GA_3_ + 6-BA.

GA_3_ + 6-BA highly increased GA_3_ content in the base part of fruits at 10 DAP and 20 DAP. Strangely, the fruits treated by PAC showed a similar pattern with GA_3_ + 6-BA application at 10 DAP and 20 DAP. The content of GA_3_ in fruits treated by GA_3_ + 6-BA was higher than that of fruits treated by PAC. At 35 DAP, there was no significant difference in GA_3_ content between GA_3_ + 6-BA-treated and control fruits, while PAC significantly reduced GA_3_ content (Figure 7a). The content of ZR in the base part of fruits at 20 DAP was significantly increased by GA_3_ + 6-BA application. PAC showed no significant influence on ZR content from control fruits at 10 DAP, 20 DAP, and 35 DAP. ZR content in the outer base part of fruits mostly was higher than that in the inner base part of fruits (Figure 7b).

PAC increased ABA content in the base part of fruits at 10 DAP. ABA content was significantly higher than GA_3_ + 6-BA-treated and control fruits at 10 DAP. At 20 DAP, the ABA content in the outer base part of fruits all exhibited lower values than that in the inner base part of fruits. At 35 DAP, three groups all showed a rapidly drop in ABA level (Figure 7c).

### Expression levels of genes related to biosynthesis and metabolism of GA, cytokinin, and ABA in the base part of fruits treated by GA_3_ + 6-BA and PAC

**Figure 8.**
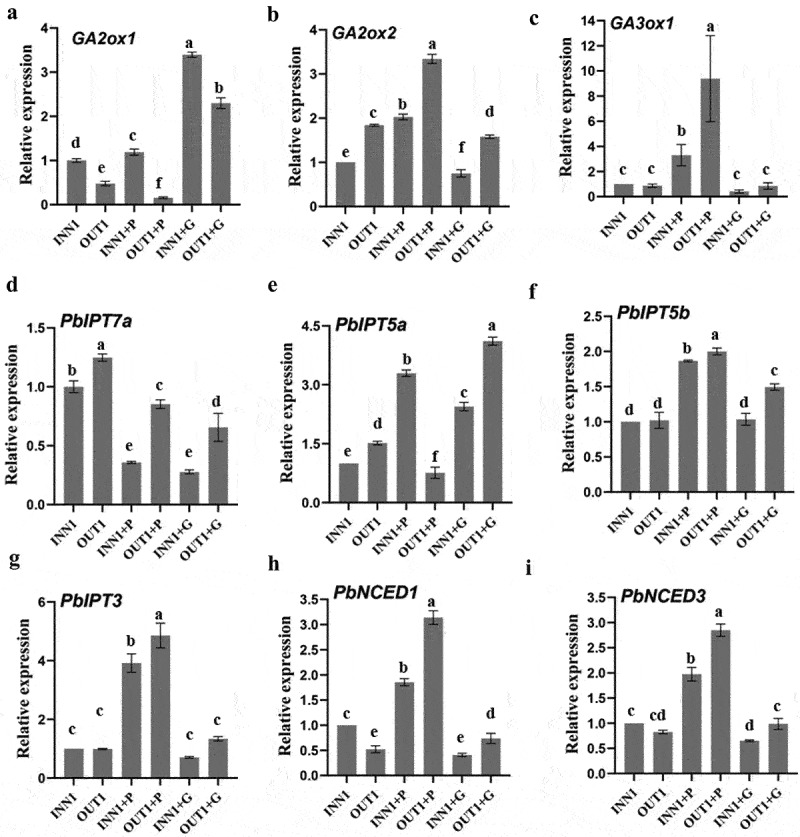
The influence of GA_3_ + 6-BA and PAC on the expression patterns of genes related to gibberellin acid (GA) metabolism, cytokinin (CTK) biosynthesis, and abscisic acid (ABA) biosynthesis at 10 days after pollination, including *PbGA2ox1* (a), *PbGA20ox2* (b), *PbGA3ox1* (c), *PbIPT7a* (d), *PbIPT5a* (e), *PbIPT5b* (f), *PbIPT3* (g), *PbNCED1* (h), and *PbNCED3* (i). Different letters above the bars indicate significant difference (Duncan’s multiple-range test, *p*≤ .05). INN1/OUT1 + P, the inner/outer base part of fruits treated by PAC; INN1/OUT1 + G, the inner/outer base part of fruits treated by GA_3_ + 6-BA.

The gene *PbGA2ox1* expression level in the outer base part of fruits all showed significantly lower values than that in inner. GA_3_ + 6-BA greatly promoted *PbGA2ox1* expression level both in the inner and outer base parts of fruits. PAC significantly suppressed *PbGA2ox1* expression level in the outer base part of fruits (Figure 8a). The gene *PbGA20ox2* possessed relatively higher expression level in the outer base part of fruits than in inner both at treated and control fruits. The *PbGA20ox2* expression level was obviously suppressed by GA_3_ + 6-BA, while it was promoted by PAC (Figure 8b). *PbGA3ox1* weakly expressed both in GA_3_ + 6-BA treated fruits and in control fruits. PAC application greatly increased *PbGA3ox1* expression level, which was significantly higher in the outer base part of fruits than that in inner (Figure 8c).

The gene *PbIPT7a* (Figure 8d) possessed significantly higher expression levels in the outer base part of fruits than that in inner both in treated and control fruits. GA_3_ + 6-BA and PAC showed no similar influence on the gene expression patterns of *PbIPT7a, PbIPT5a* (Figure 8e), *PbIPT5b* (figure 8f), and *PbIPT3* (Figure 8g). Most of them in the outer base part of fruits showed higher expression level than that in inner.

The genes *PbNCED1* and *PbNCED3* possessed a similar expression pattern both in treated and control fruits. PAC application significantly increased the expression levels of *PbNCED1* (Figure 8h) and *PbNCED3* (Figure 8i). It was consistent with the elevated ABA content in the base part of fruits treated by PAC at 10 DAP [Figure 7c). GA_3_ + 6-BA showed no significant influence on *PbNCED1* and *PbNCED3* transcription levels.

## Discussion

Yatu usually generated in the outer base part of fruit. Reference ^3^ found the similar results to us. In this study, the primitive cells of Yatu were found at 10 DAP in the outer base part of fruit. They were parenchyma cells that were characterized by small cells with more layers gathered in the outer base part of fruit compared to the surrounding cells. The 10 DAP was considered as the strongest cell division stage, which was an important time point for Yatu formation. The accelerated cell division of primitive cells with subsequent cell expansion promoted Yatu formation.

The highest content of GA_3_ and ZR was both observed at 2 DBB. There was a time lag between the high accumulation of them and the rapid primitive cells division. Reference ^23^ found that during fruit development period, cytokinin played a crucial role in stimulating cell division. Carlos et al. (2016] worked with citrus and found that GA triggered and maintained ovary-wall cell division. Therefore, we proposed the activation of primitive cells division of Yatu based on the accumulation of the relative high levels of GA_3_ and ZR during balloon period. They played an early proliferation-inducing role in the process of Yatu morphogenesis.

In most species, plant hormones were critical to control cell division and expansion.^8^ Previous studies found the high expression levels of *GA20ox* and *GA3ox* significantly promoted bioactive GAs biosynthesis in tomato and strawberry.^6,24^ The overexpression of *GA2ox* significantly decreased the content of bioactive GAs and exhibited an obvious dwarf phenotype in *solanum* and *Pyrus* rootstock.^25,26^ In this study, the lower transcription level of *PbGA2ox1* and the higher expression level of *PbGA20ox2* improved GA_3_ content in the outer base part of fruits compared to that in inner at 10 DAP. The higher transcription levels of *PbIPT7a* and *PbIPT5a* synergistically promoted the higher accumulation of ZR in the outer base part of fruits at 10 DAP compared to that in inner. Reference ^27^ found the increased expression levels of *PaNCED1* and *PaNCED3* increased ABA content in avocado fruits. In this study, the lower expression levels of *PbNCED1* and *PbNCED3* were consistent with the lower ABA content in the outer base part of fruits. We speculated that the relatively higher content of GA_3_ and ZR at 2 DBB and 10 DAP, the lower ABA content at 10 DAP promoted Yatu morphogenesis and development.

^14^ found exogenous GA and BA application regulated cell elongation and expansion through altering the microtubule array organization. Reference ^28^ found exogenous GA treatment effectively increased plant height by 20.4% via increasing epidermis cell length of the petiole. In this study, we found GA_3_ + 6-BA application significantly increased GA_3_ content at 10 DAP and 20 DAP, and increased ZR content at 20 DAP. We speculated that GA_3_ + 6-BA treatment accelerated the development process to form typical Yatu via increasing the endogenous GAs and cytokinin that promoted cell elongation.

PAC was a plant growth retardant by inhibiting GA biosynthesis. Suppressing KO, which acted in the oxidation process of ent-kaurene to ent-kaurenoic acid by the application of PAC, resulted in a reduction of GA concomitant with an inhibition on the plant growth rate,^29–31^ while the reduction could be rescued by GA application.^32,33^In this study, PAC significantly increased ABA level at 10 DAP and sharply decreased GA_3_ content at 35 DAP. We speculated that PAC application suppressed the development process to form Yatu by increasing ABA content and inhibiting GA biosynthesis that suppressed cell elongation.

In this study, we found Yatu usually generated in the outer base part of fruit, which possessed higher contents of GA_3_ and ZR at 2 DBB and 10 DAP, the lower ABA content at 10 DAP. The application of GA_3_+ BA and PAC showed promotion and suppression on Yatu formation, respectively. In short, plant hormone played an important role in regulating Yatu formation. It could be concluded that the relatively higher content of GA_3_ and ZR and lower content of ABA promoted Yatu morphogenesis and development.
